# A Program of Nutritional Education in Schools Reduced the Prevalence of Iron Deficiency in Students

**DOI:** 10.1155/2011/284050

**Published:** 2011-04-06

**Authors:** María Nieves García-Casal, Maritza Landaeta-Jiménez, Rafael Puche, Irene Leets, Zoila Carvajal, Elijú Patiño, Carlos Ibarra

**Affiliations:** ^1^Laboratorio de Fisiopatología, Centro de Medicina Experimental, Instituto Venezolano de Investigaciones Científicas (IVIC), Caracas 1020-A, Venezuela; ^2^Fundación Bengoa para la Salud y Nutrición, 8va Transversal con 7ma Avenida, Quinta Pacairigua, Altamira, Caracas 1050, Venezuela

## Abstract

The objective was to determine the prevalence of iron, folates and retinol deficiencies in school children and to evaluate the changes after an intervention of nutritional education. The project was developed in 17 schools. The sample included 1,301 children (678 males and 623 females). A subsample of 480 individuals, was randomly selected for drawing blood for biochemical determinations before and after the intervention of nutritional education, which included in each school: written pre and post-intervention tests, 6 workshops, 2 participative talks, 5 game activities, 1 cooking course and 1 recipe contest. Anthropometrical and biochemical determinations included weight, height, body-mass index, nutritional status, hematocrit, serum ferritin, retinol and folate concentrations. There was high prevalence of iron (25%), folates (75%) and vitamin A (43%) deficiencies in school children, with a low consumption of fruit and vegetables, high consumption of soft drinks and snacks and almost no physical activity. The nutritional education intervention produced a significant reduction in iron deficiency prevalence (25 to 14%), and showed no effect on vitamin A and folates deficiencies. There was a slight improvement in nutritional status. This study shows, through biochemical determinations, that nutritional education initiatives and programs have an impact improving nutritional health in school children.

## 1. Introduction

Programs on nutritional education have been widely used for teaching or reinforcing knowledge on food habits or healthy life styles in children and are considered a useful strategy to prevent the appearance of nontransmissible chronic diseases at early ages. The implementation of nutritional education programs in schools may help to inculcate in children the ability of identifying a healthy food choice for themselves [1].

It has been established that the triangulation of information amongst the teacher, the children, and the family is a useful strategy for modifying negative feeding behaviors that are contributing to the recent increase in the prevalence of overweight, obesity, hypertension, diabetes, and metabolic syndrome in children, while in the opposite extreme of the spectrum, nutritional deficits persist as important nutritional problems, especially regarding micronutrient, and vitamin deficiencies such as iron, calcium, folic acid, and vitamin A, among others [2, 3]. 

The inclusion of nutritional education into formal education programs is one of the most used and recommended strategies, mainly because the children obtain and fix the information in a easy, fun, and permanent way, but also because they act as multipliers of the information, bringing the new information to their homes to achieve, in the best case scenario, the transmission of the information to the whole family group. Some studies indicate that to obtain a better impact on changing habits on the long term, nutritional education programs must include the whole community, to assure the permanence of changes [3]. 

In general, the application of nutritional education strategies obtains a limited success when implemented as an isolated strategy. In a first stage, the simultaneous application of supplementation or fortification programs with nutritional education is the ideal approach. This is in the understanding that the 2 initially mentioned interventions should be temporary measures, while the more permanent changes in nutritional habits, are achieved with the aid of nutritional education [4]. 

 Anemia constitutes the most prevalent nutritional deficiency worldwide, especially in children and women in childbearing age. The main cause of anemia in these age groups is iron deficiency, although other nutritional deficiencies, such as folic acid, are also becoming important etiological agents [5]. Another important nutrient during growing and development periods is vitamin A, essential for vision, immunological function, development and maintenance of mucosal barriers, and so forth. 

The worldwide prevalence of anemia in preschoolers is 47.4%, and 23.1 millions of those children live in the Americas [6]. In Venezuela, the prevalence of anemia for this age group is around 30% [5, 7, 8], although a study performed in groups from the marginal socioeconomic strata reported a 75% prevalence of anemia [9]. Folic acid deficiency is also high for preschool children reaching 31% in a National survey [10, 11]. Vitamin A deficiency has a prevalence of 33.3%, affecting 190 millions of children, half of which live in the Americas [6]. In Venezuela, there are few documented reports on vitamin A deficiency, which indicate a prevalence of 25–30% in children from low socioeconomic strata of the population [12, 13].

Due to the importance of these 3 nutrients for growing and development and to the higher susceptibility to suffer from these deficiencies during infancy and childhood, the objective of this study was to determine the prevalence of iron, folates, and vitamin A deficiencies in school children from 6 to 14 years and to evaluate the changes in these parameters after an intervention of nutritional education.

## 2. Materials and Methods

The project “Iron deficiency and anemia” was approved by Fundacion Bengoa Review Board and developed in 17 schools from 3 Venezuelan States (Aragua, Lara, and Táchira) located in the northwest region of the country. The sample was constituted by scholars from 6 to 14 years of age, selected from the inscription lists of children and adolescents to the schools of the 3 zones, during September 2007 to November 2008. 

The sampling was probabilistic, in which all children from all the schools selected had a known and not null probability of being part of the sample. The sample size was 1,301 children (678 males and 623 females), and a subsample of 480 individual, were randomly selected for blood sampling and for biochemical and anthropometric determinations before and after the intervention of nutritional education. The first blood drawing had 2 objectives: 1 to determine the prevalence of iron, folic acid, and vitamin A deficiencies in the zone and 2 to establish a baseline or time 0, before the intervention. The second blood draw, 6 months later, allowed to verify any changes in the biochemical parameters after the intervention.

Parents, children, and teachers received detailed information about the procedure and the protocol, and an informed consent was signed by all participants. After inclusion in the protocol, a detailed record with personal data, medical, and nutritional records was filled for each participant.

### 2.1. Nutritional Education Intervention

The nutritional education program was initiated with a written test to explore the previous nutritional knowledge of teachers, and from those results the intervention was designed. The objective was to educate teachers about the importance of a proper nutrition with emphasis on anemia and iron deficiency prevention, so they can act as multipliers transferring the information to their students.

The program contents included basic concepts in food and nutrition, how to obtain a balanced meal, anemia and iron deficiency as public health problems, growth and development according to what we eat, balanced and varied meals, food iron sources, food iron absorption enhancers and inhibitors, and menu preparation. The program in each school consisted in 6 workshops, 2 participative talks, 5 game activities, 1 cooking course, and 1 recipe contest designed and directed to teachers and/or pupils. In the workshops, different techniques were applied to obtain a harmonious and participative ambient in which interventions were encouraged and the knowledge was obtained through “learning by doing.” The teachers developed original educative strategies (role-playing, songs, poetries, puppets, etc.), expositions, and participative talks. Each teacher received a set of technical notes, with the contents to teach and the transfer of knowledge was evaluated by direct supervision of the teacher, by the activities performed, the techniques used during the activities, and also by another written test at the end of the intervention.

### 2.2. Anthropometric and Biochemical Analysis

The subsample of children and adolescents was measured in light clothes at the beginning and end of the nutritional education intervention, and weight and height were recorded by trained personnel. The measuring error was less than 1%, within expected limits. The classification according to nutritional status was performed by the combination of indicators: weight-age, height-age, and body mass index (BMI-) age. The data was grouped according to presumptive diagnosis as deficit (lower than percentile 3), at risk of deficit (≥ percentile 3 and < percentile 10), normal (≥ percentile 10 and ≤ percentile 90), at risk of excess (> percentile 90 and ≤ percentile 97), and excess (> percentile 97). A 24 h food recall interview was also performed in children and adolescents from 10 to 14 years of age.

To the same group, 2 blood samples were taken at the times mentioned, to perform hemoglobin and hematocrit in blood and to determine serum concentrations of ferritin, folates, and retinol. Each sample consisted in 5 mL of blood taken from the antecubital vein of the arm after cleaning of the zone with isopropyl alcohol. From this, 4 mL were used to obtain serum and 1 mL was treated with EDTA for hemoglobin and hematocrit determinations.

Due to logistic problems and improper blood conservation and transport, it was not possible to determine hemoglobin and perform anemia prevalence analysis. Therefore, the results reported in this study include hematocrit (determined in a microcentrifuge at the place of sampling), serum ferritin, folates, and retinol concentrations and the prevalence of iron, folates, and retinol deficiencies.

Serum samples were obtained by centrifugation of blood samples, within 4 hours of extraction. Serum was kept at −30°C and protected from light until analysis.

Ferritin determinations were performed by an immunoenzymatic assay [14] developed and validated in our laboratory. Polystyrene microtiter plates were coated with monoclonal antibodies to human ferritin. In each assay, ferritin standards and unknown sera were added and incubated with the same monoclonal antibody conjugated with horseradish peroxidase. The amount of enzyme retained in each well was measured by adding o-phenylenediamine dihydrochloride (OPD) and H_2_0_2_, and the reaction stopped by adding sulfuric acid. The absorbance in each well was measured spectrophotometrically, and ferritin concentration was calculated from a standard curve.

Serum retinol was assayed by high-performance liquid chromatography (HPLC) according to the method of Chow and Omaye [15]. Briefly, 100 *μ*L of serum were extracted with heptane containing BHT, dried under a nitrogen stream, and suspended in methanol for separation in an HPLC system (Waters corporation, Mildford MA) with a Bondapack C18 column (3.9 × 300 mm), using 100% methanol as mobile phase at 0.8 mL/min. Detection was carried at 296 nm and retinol concentrations were calculated from a standard curve using Empower software from Waters. 

The microbiological method for the determination of serum folates was an adaptation by the Centers for Diseases Control and Prevention (Atlanta, USA) of the method described by O'Broin and Kelleher [16] and Molloy and Scott [17]. It was based in the folate-dependent growth of a strain of *Lactobacillus.* Serum samples and bacteria were diluted in a complete nutritive medium devoid of folates. The growing of the bacteria was dependent of the presence of folates in the sample and measured by the turbidity developed in the wells at 590 nm, comparing against a standard curve.

The cutoff points used were for iron deficiency, a serum ferritin level <10 *μ*g/L [6], for vitamin A, deficiency was classified as severe when serum concentration was <10 *μ*g/dL, and moderate if <20 *μ*g/dL [18]. For folic acid deficiency, it was severe for serum concentrations below 6.7 nmol/L, and moderate between 6.7 and 13.2 nmol/L [10, 11].

### 2.3. Statistical Analysis

Samples were analyzed for hematocrit and serum concentrations of ferritin, folates, and retinol and expressed as mean ± SD and % of prevalence of the respective deficiency. The analysis of data by sex and geographic location was performed by ANOVA with Bonferroni as a posttest. For comparisons of groups before and after nutritional education intervention, paired *t*-test was used. Differences were considered as significantly different, when *P* < .05.

## 3. Results

Table 1 shows the anthropometric and hematological characteristics of the children studied. At the moment of inclusion in the protocol, the group presented normal ranges, although close to lower limits, of hematocrit, ferritin, and retinol concentrations. The mean concentration of folates was below the cutoff point of 13.2 nmol/L, indicating a moderate folate deficiency in the population studied. The table also shows that after the nutritional education intervention, all parameters analyzed, except for retinol and folates, increased significantly (*P* < .05).

When classifying children according to their nutritional status before and after intervention, the percentages diminished for deficit (from 17.1 to 16.3%), at risk of deficit (from 14.2 to 12.9%), and at risk of excess categories (from 5.0 to 2.5%), while the excess category increased from 13.7% to 15.0% (Table 2). Some of the children classified as at risk of excess and excess after the intervention were in reality children with chronic undernutrition in process of weight recovery, but with a chronically diminished height. 

There were no differences in the percentages of excess and deficit between the 3 states studied, and according to the 24 h food recall questionnaires, the diets were hypocaloric, with low consumption of fruit and vegetables, high consumption of soft drinks and snacks and low or no physical activity. Red meat was the main source of heme iron consumed by 57% of the children (54 g/day). Consumption of soft drinks and natural fruit juices was similar (47%), and coffee and tea was consumed by 22% of the sample. After intervention, there was a slight improvement in fruit consumption, based on the 24 h food recall questionnaires. 

The intervention of nutritional education produced a significant increase in hematocrit and ferritin concentration, while retinol and folate concentrations showed no significant changes. There were also no significant differences by gender, and as shown in Table 3, when distributed by state, children from Táchira presented a better nutritional condition in terms of hematocrit (data not shown), ferritin, retinol, and folates status than children from Lara. Children from Aragua State showed no difference compared to Táchira or Lara, probably as a consequence of the smaller sample size.

**Figure 1:**
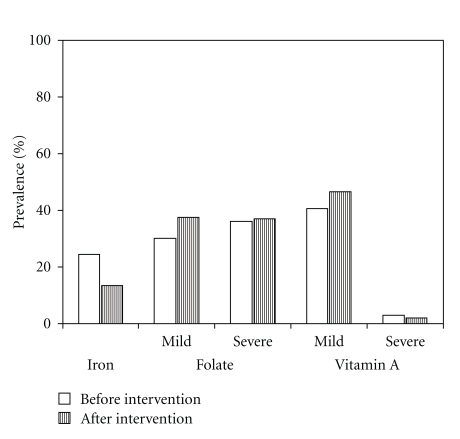
Prevalence of iron, folate, and vitamin A deficiencies in children, before and after the intervention of nutritional education in schools from Aragua, Táchira, and Lara States, Venezuela. (*n* = 427).

The prevalence of iron deficiency was 25% at the beginning of the study and was significantly reduced to 14.3% after the intervention (Figure 1). The prevalence of severe and moderate folate deficiencies affected 39% and 36% of the sample, respectively, to reach approximately 75% of the sample being affected by some degree of folate deficiency. This degree of prevalence was not affected by the nutritional education intervention. The prevalence of serum retinol deficiency was severe in less than 3% of the cases, but moderate deficiency affected more than 40% of children. As with folates, retinol levels were not significantly affected by the intervention.

## 4. Discussion

The results presented in this work show a high incidence of micronutrient deficiencies in scholars from 17 schools located in 3 noncontiguous states of Venezuela, which could be indicating a problem of hidden hunger, since anthropometrical parameters were within normal ranges. 

There was high prevalence of iron, folates, and vitamin A deficiencies in children and adolescents. The prevalence of severe folic acid deficiency affected 39% of the sample and requires immediate action, which can be achieved by a supplementation program in schools. The situation of this vitamin in Venezuela has been previously reported [10, 11], and at this point it probably could be better combated by a combined strategy, consisting in supplementation, food fortification, and nutritional education in a first stage.

The prevalence of moderate vitamin A deficiency affected 40% of the sample. If this data were extrapolated to general population, this would constitute an important Public Health problem. Regarding iron deficiency, the prevalence was 25% at the beginning of the study, a percentage similar to previously reported data for these age groups [5]. Unfortunately, due to the problems with blood and the impossibility to obtain reliable results on hemoglobin concentrations, we were unable to determine if the intervention had effects on anemia prevalence. However, a very reliable indicator of red cell volume, hematocrit, was significantly increased after the nutritional education program. 

The most efficacious strategies to combat nutritional deficiencies are food fortification and supplementation programs, although it is generally recognized that nutritional education should always accompany those initiatives and also that education is the most fundamental and permanent strategy to achieve changes in food habits and to obtain a balanced nutrition that includes all the required nutrients during the different life stages [19]. However, it has been pointed out that the success of educative programs is dependent on the enthusiasm and commitment of teachers to include the suggested strategies and also of their interest in master the knowledge required and the time to implement those new contents [20].

Limitations of educational campaigns include a non-immediate, long-term effect or impact as well as a limited penetration that does not guarantee complete access or coverage [21]. Also, the impact of educational interventions is usually measured by tests that evaluate if the children received and understood the message [19, 22]. The reports in the literature about measuring biochemical parameters to evaluate the impact of a nutritional education intervention are scarce, but these evaluations are a direct measure that “the message was received, understood and put into practice,” a conduct that probably would benefit not only the child or the person receiving the information, but also the whole family group.

The analysis of the impact of the nutritional intervention performed was measured not only by direct observation and supervision of activities and games, where teachers and pupils could demonstrate the acquisition of the knowledge, but also by the significant reduction in iron deficiency prevalence, measured as serum ferritin, after the intervention. There were, however, no changes in serum retinol and folate concentrations, both nutrients with high prevalence of deficiency. The lack of effect on those 2 nutrients could be due, in part, to the fact that the intervention was focused on iron and iron rich foods sources. Also, the limited access to food of animal origin and the low vegetable and fruits consumption could account for the lack of effect observed. In this group, precooked corn flour (fortified with iron) was the most consumed food (2 corn breads called “arepas” per day, 100 g/day) and constituted 96% of the iron in the diet, followed by grains. Furthermore, consumption of performed vitamin A, which should come from animal sources, was limited in this group, as well as a provitamin A carotenoids, also a good source of folates. 

This study shows, through biochemical determinations, that nutritional education initiatives have an impact improving nutritional health in children. However, the permanence in time of such habit modifications that assure the maintenance of the biochemical changes achieved requires more ample interventions that involve the whole community, as well as the treatment of other external factors that affect the appearance of nutritional deficiencies [3, 20]. 

The combination of strategies (education, supplementation, and/or food fortification) is probably the most effective approach to combat micronutrient deficiencies, especially severe deficiencies as in the case of folic acid in this study. It would be desirable to perform strategies in nutritional education at school level, with measuring the impact through biochemical variables at the family group level. Also, existing educational campaigns should be reinforced to encourage consumption of vegetables, fruits, and other sources of folates and vitamin A.

##  Conflict of Interests

They also declare that they have no conflicto of interests.

## Figures and Tables

**Table 1:** Changes in anthropometric and hematological parameters in children before and after the intervention of nutritional education in schools from Aragua, Táchira, and Lara States, Venezuela. (*n* = 427).

	Before intervention	After intervention
Age (years)	8.68 ± 2.31	9.44 ± 2.44*
Weight (Kg)	28.47 ± 9.69	31.12 ± 12.11*
Height (cm)	127.49 ± 13.80	131.40 ± 15.88*
Hematocrit (%)	37.42 ± 3.32	38.57 ± 2.90*
Ferritin (*μ*g/L)	18.04 ± 12.00	22.56 ± 15.82*
Serum retinol (*μ*g/dL)	22.09 ± 6.61	20.73 ± 5.80
Serum folate (nmol/L)	11.74 ± 7.19	10.58 ± 8.80

*Statistically significant difference between before and after intervention (*P* < .05).

**Table 2:** Classification of children according to nutritional status, by combination of indexes before and after nutritional education intervention.

		Before intervention		After intervention	
Categories*	Total	*n*	%	*n*	%
Deficit	80	41	17.1	39	16.3
At risk of deficit	65	34	14,2	31	12.9
Normal	248	120	50.0	128	53.3
At risk of excess	18	12	5.0	6	2.5
Excess	69	33	13.7	36	15.0

Total	480	240	100	240	100

*The data was grouped according to presumptive diagnosis as deficit (lower than percentile 3), at risk of deficit (≥ percentile 3 and < percentile 10), normal (≥ percentile 10 and ≤ percentile 90), at risk of excess (> percentile 90 and ≤ percentile 97), and excess (> percentile 97).

**Table 3:** Changes in ferritin, retinol, and folate concentrations in children before and after the intervention of nutritional education in schools from Aragua, Táchira, and Lara States, Venezuela. Classification by State of precedence.

		Ferritin (*μ*g/L)		Serum retinol *μ*g/dL		Serum folate nmol/L	
	*n*	Before intervention	After intervention	Before intervention	After intervention	Before intervention	After intervention
Aragua	69	12.00 ± 10.17^a^	24.33 ± 18.34*	20.66 ± 6.72^a,b^	17.17 ± 3.69^a^	7.43 ± 4.99	10.34 ± 8.90^a,b^
Táchira	179	21.06 ± 12.81^b^	23.60 ± 16.48	23.72 ± 5.95^b^	22.45 ± 5.88^b^	12.99 ± 7.24	12.16 ± 9.29^b^
Lara	179	16.52 ± 10.62^a,b^	20.88 ± 13.90	20.50 ± 6.91^a^	20.48 ± 5.74^b^	11.21 ± 9.35	9.19 ± 8.07^a^

Total	**427**	16.53 ± 13.17	22.94 ± 16.24*	21.66 ± 6.64	20.09 ± 5.10	10.54 ± 7.19	10.56 ± 8.75

*Statistically significant difference between before and after intervention (*P* < .05).

^a^Different superscript letters, statistically different between states (*P* < .05).
